# A Narrative Review: Gingival Stem Cells as a Limitless Reservoir for Regenerative Medicine

**DOI:** 10.3390/ijms23084135

**Published:** 2022-04-08

**Authors:** Luigia Fonticoli, Ylenia Della Rocca, Thangavelu Soundara Rajan, Giovanna Murmura, Oriana Trubiani, Stefano Oliva, Jacopo Pizzicannella, Guya Diletta Marconi, Francesca Diomede

**Affiliations:** 1 Department of Innovative Technologies in Medicine & Dentistry, University “G. d’Annunzio” Chieti-Pescara, Via dei Vestini, 31, 66100 Chieti, Italy; luigia.fonticoli@unich.it (L.F.); ylenia.dellarocca@unich.it (Y.D.R.); giovanna.murmura@unich.it (G.M.); oriana.trubiani@unich.it (O.T.); oliva.stefano@hotmail.com (S.O.); francesca.diomede@unich.it (F.D.); 2 Department of Biotechnology, Karpagam Academy of Higher Education, Coimbatore 641 021, India; drsoundararajan.t@kahedu.edu.in; 3 Ss. Annunziata Hospital, ASL 02 Lanciano-Vasto-Chieti, 66100 Chieti, Italy; 4 Department of Medical, Oral and Biotechnological Sciences, University “G. d’Annunzio” Chieti-Pescara, Via dei Vestini, 31, 66100 Chieti, Italy; guya.marconi@unich.it

**Keywords:** human gingival mesenchymal stem cells, regenerative medicine, stem cells niche, biomaterials

## Abstract

The gingival tissue can be collected in an easy way and represent an accessible source to isolate gingival-derived mesenchymal stem cells (GMSCs). GMSCs are a subpopulation of dental-derived mesenchymal stem cells that show the mesenchymal stem cells (MSCs) features, such as differentiation abilities and immunomodulatory properties. Dental-derived stem cells are also expandable in vitro with genomic stability and the possibility to maintain the stemness properties over a prolonged period of passages. Moreover, several preclinical studies have documented that the extracellular vesicles (EVs) released from GMSCs possess similar biological functions and therapeutic effects. The EVs may represent a promising tool in the cell-free regenerative therapy approach. The present review paper summarized the GMSCs, their multi-lineage differentiation capacities, immunomodulatory features, and the potential use in the treatment of several diseases in order to stimulate tissue regeneration. GMSCs should be considered a good stem cell source for potential applications in tissue engineering and regenerative dentistry.

## 1. Introduction

Recently, preclinical and clinical studies have evidenced the vital role of MSCs in tissue homeostasis and their potential use in tissue engineering and regenerative field as well as in cell-based regenerative treatments to cure a variety of autoimmune and inflammatory illnesses and regenerate injured tissue.

The regenerative beneficial effects of MSCs are related to their multipotent abilities, but recently, novel evidence reported that MSC-mediated curative impacts are due also to their great immunomodulatory/anti-inflammatory properties and to their secretome features, including growth factors, cytokines, hormones, miRNAs, and other bioactive soluble factors [1].

Adult bone marrow was the initially identified source of MSCs (BM-MSCs) [2]. In the last few decades, MSCs’ populations were isolated additionally from many other tissues and organs, such as adipose tissue, periosteum, trabecular bone, synovium, skeletal muscle, and teeth [3].

The oral cavity has proved to be an important alternative source of MSCs. The oral MSCs exhibit similar multipotency characteristics compared to other MSCs; moreover, they are easily accessible, show genomic stability, and, in opposition to BM-MSCs, they have a faster proliferation rate [4,5].

The areas of the oral cavity from which MSCs can be obtained are: dental pulp, periodontal ligament, apical papilla, dental follicle, and gingival tissue [6,7] Among these, periodontal ligament stem cells (hPDLSCs) and hGMSCs are the cells with easier accessibility, representing two important alternative to classic MSCs [8].

The human gingiva is a unique masticatory keratinized mucosal tissue and an essential component of the periodontium containing hGMSCs, cells that can be easily isolated from human biopsy fragments through poorly invasive techniques [9,10]. hGMSCs were first isolated by Zhang et al. from waste gingival tissue of patients in good health condition and with a healthy periodontium who underwent orthodontic treatments [11].

These cells evidence stable phenotype and telomerase activity in long-term cultures and are not tumorigenic. In addition, they show self-renewal ability and are capable of originating connective tissue-like structures in vivo [12].

Furthermore, hGMSCs are well known for their multipotency and their capacity to differentiate in different cell types, including, but not limited to, osteocytes, odontoblasts, adipocytes and myocytes (Table 1).

These features, in addition to hGMSCs’ anti-inflammatory properties and their paracrine activity mediated by the EVs, make these cells a promising resource in the clinical application of tissues regeneration [21].

In recent years, a lot of research has been focused on new cell-free therapies with EVs and conditioned medium (CM) obtained from hGMSCs. EVs contain cellular products that need to be transported through the biological barriers to promote cell communication. At the same time, EVs show similar properties, as well as similar regenerative potential, to hGMSCs [22].

The current review aims at reporting the features and biological properties of hGMSCs and reviewing the promising role played by hGMSCs and their cellular products in therapeutic applications in the regenerative medicine and tissue engineering field.

## 2. Adult Mesenchymal Stem Cells

The discovery of MSCs occurred in the second half of 1900. In 1966, Friedenstein and his team discovered the multipotency characteristic of these cells, but only in 1991 did Caplan et al. coin the term MSCs. In recent decades, numerous debates have been raised regarding the nomenclature of MSC; some researchers prefer to define these cells as stromal or medicinal signaling cells to underline their secretory capacity. However, their self-renewal capacity justifies the definition of stem cells [23]. As reported in 2006 by Dominici et al., the Mesenchymal and Tissue Stem Cell Committee of the International Society for Cellular Therapy listed the minimum criteria for defining MSCs with the aim of favoring a more equable characterization of MSCs. Specifically, MSCs must have the ability to grow and proliferate on plastics when placed in in vitro cultures and to differentiate into different cell lineages such as osteogenic, chondrogenic and adipogenic lineages. They possess a fibroblast-like morphology, mesenchymal markers such as CD105, CD90, and CD73, and are negative for endothelial and hematopoietic surface markers CD11b, CD19, CD79α, CD31, CD34, CD45, and HLA-DR antigen [24].

In addition, oral MSCs can maintain tissue homeostasis and regenerate damaged tissues, reduce the immune response and carry out anti-inflammatory action during the healing process [25].

MSCs display paracrine functions that are involved in the modulation of inflammatory processes and immune responses. Indeed, MSCs play a role in adaptive and innate immunity through the suppressive effect on T cells, B cells, dendritic cells, and natural killer cells [26].

Silvestro et al. reported the immunomodulatory and trophic support capacities of MSC-derived conditioned medium (CM) delivered in the damaged site of a human spinal cord injury. The immunomodulatory properties were due to the growth factors present in the CM, such as brain-derived neurotrophic factor (BDNF), glial-derived neurotrophic factor (GDNF), vascular endothelial growth factor (VEGF), and Nerve growth factor (NGF). The anti-inflammatory capacity was linked to the modulation of cytokines such as tumor necrosis factor-α (TNF-α), interleukine-1β (IL-1β), interleukine-6 (IL-6), interleukine-4 (IL-4), interleukine-2 (IL-2), and interleukine-12 (IL-12) [27].

## 3. Oral Mesenchymal Stem Cells

The human oral cavity represents an excellent source of stem cells due to its easy accessibility. As previously reported, different types of MSCs are distinguished in relation to their site of origin: human dental pulp stem cells (hDPSCs), human exfoliated deciduous teeth stem cells (hSHEDs), human periodontal ligament stem cells (hPDLSCs), human apical papilla stem cells (hSCAPs), human dental follicle stem cells (hDFSCs) and hGMSCs [28]. The first MSCs isolated from the tooth were hDPSCs. These cells were able to differentiate into osteocytes, chondrocytes and adipocytes [29].

Successively, hSHEDs and hSCAPs have been demonstrated to have similar differentiation capabilities as hDPSCs but with higher proliferative activity than hDPSCs and BM-MSCs. Previous works reported that hSCAPs have good regenerative potential and are able to produce dentin in vivo [30]. On the other hand, hDFSCs, isolated from the third molar, can differentiate into cementoblasts and are able to produce cement and bone tissue in vitro. Instead, hPDLSCs can differentiate into many cell types and appear to be involved in the regenerative processes of the periodontium (Table 2) [31].

hGMSCs, derived from the connective tissue of the gingiva, can be differentiated into two cell types with different origins. During embryonic development, a population of multipotent stem cells are present in the neural crest level. From this area, different tissues originate, including neural tissues, cartilage, bone, and teeth. Several studies on animal models confirm the presence of a reservoir of stem cells with neural origins in both embryonic and postnatal tissues. Cells with neural origins have also been identified in tissues of the oral cavity, such as gingiva, dental pulp, apical papilla, dental follicle, and periodontal ligament [39]. Specifically, in gingival tissue, neural crest-derived stem cells (N-hGMDCs) have been identified that represent the 90% of all hGMSCs and mesoderm-derived stem cells (M-hGMSCs), which are the other 10% [40]. Both N-hGMDCs and M-hGMSCs are multipotent cells and express the same surface markers typical of MSCs. However, they are distinguished by some characteristics. The N-GMSCs express, both during embryonic development and in adult tissue, neural crest-associated genes Sox9, Twist1, Snail, Myc, Ets1, Crabp1, Epha2, and Itgb1 [41]. Furthermore, they show a greater differentiation capacity toward the neurogenic and chondrogenic phenotype compared to M-hGMSCs. These properties make N-hGMDCs valid alternatives for the regeneration of neural tissue and cartilage [42]. On the other hand, no differences in osteogenic and adipogenic differentiation has been shown in the two cell types [43].

Overall, both hGMSCs show an excellent proliferative and migratory capacity, have a stable phenotype, and are involved in tissue repair and homeostasis. For these reasons, they represent excellent candidates for regenerative medicine therapies.

## 4. Anti-Inflammatory and Immunomodulatory Properties of hGMSCs

The gingival tissue is often the site of infections, frequently of bacterial origin such as gingivitis, which can cause alterations in the structure and functionality of the tissue. At the same time, it is widely reported that hGMSCs obtained from healthy gingival tissue have similar characteristics to hGMSCs isolated from inflamed gingiva. The cells of inflamed tissue have the ability to differentiate in vitro into adipogenic, osteogenic and chondroigenic phenotypes, express the same superficial markers, and show the same immunomodulatory properties of hGMSCs obtained from fragments of healthy gingiva. Furthermore, some previous studies have reported that a series of subcutaneous ectopic transplants of hGMSCs, obtained from both inflamed and non-inflamed tissues, are capable of generating connective tissue similar to that of healthy gingiva. As a result, inflamed gingival tissue could also be considered a valid source of hGMSCs. Thus, both hGMSCs appear to play an important role in tissue regeneration [44].

hGMSCs exhibit anti-inflammatory and immunomodulatory properties that modulate the innate and adaptive immune responses. During the innate immune response, hGMSCs modulate the activation of immune cells, such as macrophages, mast cells, dendritic cells, and natural killer cells (Table 3).

In detail, hGMSCs stimulate the polarization of macrophages and promote the anti-inflammatory M2 phenotype by improving the secretion of anti-inflammatory cytokines such as IL-10 and decreasing the expression of IL-6 and TNF-α. Furthermore, as confirmed by previous in vivo and in vitro studies, hGMSCs decrease the degranulation of mast cells employed in the immune response. They reduce the expression of mature dendritic cells markers and the production of some proinflammatory cytokines. hGMSCs also express their immunomodulatory action on cells involved in adaptive immunity, particularly T helper cells. The hGMSCs reduce the proliferation of peripheral blood mononuclear cells and are involved in the suppression of B cells’ proliferation and differentiation. The hGMSCs, under inflammation conditions, take part in the inflammatory mechanisms of tissue healing through their anti-inflammatory action. The cellular receptors involved in the innate and adaptive immune response are the Toll-like receptors (TLRs). These receptors recognize pathogens through the molecular pattern associated with specific pathogens (PAMPs) and start the immune response [50]. As reported by previous in vitro studies, hGMSCs, in the absence of an inflammatory stimulus, express the basal level of TLR 1, 2, 3, 4, 5, 6, 7, and 10, while they exhibit a higher expression of all TLRs except TLR 6 when cultured with medium supplemented with inflammatory cytokines, such as IL-1β, interferon-γ (IFN-γ), TNF-α, and IFN-α. This expression confirms that hGMSCs recognize PAMPs and start their anti-inflammatory action. For this reason, they represent an ideal study model for the identification of new treatments to apply for inflammatory diseases, such as periodontitis [51,52].

## 5. Extracellular Vesicles (EVs) and Secretome Derived from hGMSCs

In recent decades, cell-free therapies are attracting the attention of the scientific community due to their reduced costs in contrast to cell-based therapies, lower risk of rejection or infections, and the absence of uncontrolled differentiation that can occur with cells treatments [53]. It is largely reported that MSCs often act through paracrine mechanisms, playing a pivotal role in cell communication. In particular, the secretome of MSCs shows regenerative potential similar to MSCs and, consequently, is often used in cell-free tissue regeneration therapies [54].

The CM obtained from MSCs’ cultures is rich in proteins, cytokines, nucleic acids, and growth factors, which take part in the regenerative processes. Based on the literature, CM obtained from BM-MSCs was involved in bone regeneration and showed a higher regenerative power than MSCs. CM recruits endogenous MSCs and accelerates the bone regeneration process. An analogous mechanism was also observed in CM obtained from adipose tissue stem cells [55]. Similar properties were also found in the CM obtained from hGMSCs (hGMSCs-CM). As reported by Qiu J. et al., the hGMSCs-CM enhances the periodontal tissue regeneration in rats with periodontal defects, confirming the key role of hGMSCs-CM in bone regeneration and repair processes [56]. hGMSCs-CM show a neuroprotective role and can be used as a potential treatment for degenerative motor neuron diseases. As confirmed by in vivo motor neuron degenerative disease models, hGMSCs-CM reduced inflammation, controlled apoptosis process and oxidative stress and decreased cell death in damaged motor neurons [57]. Among cellular secretion products, EVs have become the main subject of study in the regenerative medicine field. EVs show the same characteristics and similar therapeutic potential as the cells from which they are released; in addition, they can be preserved for long periods without losing their characteristics [58].

It is widely reported that EVs produced by MSCs represent a good alternative for the treatment of skin disorders, autoimmune and inflammatory diseases, oral and craniofacial disorders nerve regeneration, spinal cord injury, and bone and cartilage regeneration [39].

Recent research has focused on EVs derived from hGMSCs, especially for the easy accessibility of these cells. hGMSCs produce two different populations of EVs between 100 and 1200 nm that express the markers CD9, CD63, CD81, and TSG101 and show similar regenerative potential as hGMSCs [59]. EVs are involved in carrying nucleic acids, enzymes, and transcription factors towards target cells. Specifically, these vesicles interact with target cells binding their surface receptors, merging their membrane, and transferring lipids and proteins inside their cytoplasm. Therefore, EVs act as a vehicle in the cellular exchanges where they are involved in the horizontal transfer of mRNA to target cells to produce specific proteins. The transcriptomic analyses of hGMSCs-EVs reveal mRNAs that code for proteins involved in the inflammatory response, bone regeneration, neuronal differentiation processes, and the regulation of angiogenesis, such as interleukins, TGF-β, BMPs, GDFs, Wnt, VEGF, FGF, and neurotrophins. In addition, it is reported that EVs contain non-protein-coding RNAs (ncRNAs) involved in the modulation of cellular processes through the regulation of chromatin structure, transcription, splicing, and translation processes [60].

A variety of molecules and cellular products derived from hGMSC-EVs allows these vesicles to play a key role in tissue regeneration processes.

hGMSC-EVs’ role in bone tissue regeneration is reported by Diomede et al., who show how hGMSCs, in combination with their EVs and three-dimensional (3D) engineered scaffolds composed by poly-lactide acid (PLA), promote osteogenesis and angiogenesis in rat models with cranial bone defects [21,61].

In a recent work published by Shi H.Z et al., it is reported that hGMSCs-EVs can reduce the levels of p21, mTOR/pS6, IL-6, and TNF-α, the oxidative stress of endothelial cells and skin fibroblasts in aged mice, suggesting their use in aging-related skin and vascular dysfunctions therapies [62]. The anti-inflammatory action carried out by hGMSC-EVs is widely reported and makes these vesicles also suitable for the treatment of inflammatory diseases [45]. In a microenvironment with high lipid content and with macrophages activated by lipopolysaccharide (LPS) from *Escherichia coli*, hGMSC-EVs appear to limit the accumulation of lipids, reduce the expression of pro-inflammatory molecules such as TNF-α, IL-6, and IL-1β and promote the polarization of macrophages in the M2 anti-inflammatory phenotype [63].

The promising opportunity of using GMSCs-EVs as an alternative to cell therapies is also underlined by their role in the treatment of autoimmune diseases. Their application in these diseases is comparable to or better than those carried out with cell therapies. In mice models with collagen-induced arthritis (CIA), both the treatments with hGMSCs or hGMSCs-EVs reduced the expression of IL-17A and promoted the expression of IL-10. However, the EVs treatments showed greater effects in preventing bone erosion [64].

The large number of works on the therapeutic application of EVs produced by hGMSCs and other MSCs of different origins confirm that these cellular products represent a valid and promising alternative to cell-based therapies in tissue regeneration [57].

## 6. hGMSCs and Regenerative Medicine

Over the years, the use of stem cells associated with the most varied types of three-dimensional engineered scaffolds for the creation of implants capable of restoring damaged organs and tissues has led to the rapid advancement and modernization of personalized regenerative medicine. 

Several studies on tissue engineering have led to the development of regenerative therapies that combine scaffolds, which provide structural support, and cells [14,65]. The scaffolds used in tissue regeneration are ceramics, metals, and polymers able to promote proliferation, migration, and differentiation of the cells in contact with the scaffold. The scaffold, in addition to providing support for cell growth, can be treated and modified in order to promote the release of molecules, such as osteogenic and angiogenic factors, involved in the regeneration of tissue [66].

hGMSCs are finding applications in cell therapies, cell-free therapies, and tissue regeneration associated with the scaffolds.

Many in vitro and in vivo studies on hGMSCs confirmed the role of these cells in the regeneration of different tissues, such as bone, muscles, nerves, and skin. hGMSCs find great applications in orthopedic treatments and tissues regeneration of oral craniofacial disorders [67]. As reported by Wang X. et al., mineralized collagen-GAG scaffolds without the addition of other growth factors can improve the hGMSCs’ ability to differentiate toward the osteogenic lineage, modulate the remodeling of the matrix, and increase new mineral deposition, confirming the central role of these cells in bone regenerative processes (Table 4, Table 5, Table 6 and Table 7) [68].

The use of these cells in combination with three-dimensional structures is already known for treatments of calvaria defects, periodontal disease, and alveolar bone defects.

hGMSCs encapsulated in RGD-coupled alginate microspheres enhance bone regeneration in mice models with calvaria defects [84]. Furthermore, the same combination of RGD-coupled alginate scaffolds with hGMSCs improves myogenic regeneration when transplanted subcutaneously into immunocompromised mice [20]. The role of hGMSCs in the regeneration of muscle diseases is confirmed by their addition to the porcine small intestinal submucosa extracellular matrix (SIS-ECM). Indeed, the hGMSCs/SIS-ECM combination improves soft tissue healing and muscle tissue regeneration in rat models with critical size myomucosal tongue defects [85]. As reported by Shafiee et al. in a recent work, hGMSCs, when seeded in a polycaprolactone scaffold (mPCL), enhance wound closure with a consequent reduction in scar formation in rat models with excisional wounds [74]. The hGMSCs are also involved in the anatomical and functional repair of damaged facial nerves, as demonstrated by the differentiation of hGMSCs associated with hydrogel scaffolds into neuronal cells [16,81]. Some research underlines that injected hGMSCs have anti-inflammatory effects [82], show immunomodulatory activities [86], and promote tissues repair, e.g., by improving the wound healing process in skin [87].

As reported earlier, EVs and CM produced by hGMSCs represent new and promising alternatives to therapies that involve the use of cells. The absence of the cellular component, in addition to the easy isolation and storage of these cellular secretions, makes cells-free therapies safer than cell-based therapies. To date, their use for tissue regeneration on animal models has shown their role in the regeneration of different tissues, such as bone and neuronal tissue [60].

## 7. Disadvantages and Limitations on hGMSCs Practical Use

The use of hGMSCs in regenerative medicine and cells therapies shows many advantages and some disadvantages related to the cell features. One of the main features that make hGMSCs a potential alternative to the classic MSCs source, such as BM-MSCs, is their easy accessibility from gingival tissue. The hGMSCs’ isolation technique is a non-invasive procedure and takes place starting from gingival tissue collected during a standard dental procedure. For this reason, it is possible to obtain a large number of hGMSCs for clinical applications in a short time. In addition, these cells, if cultured in an in vitro model, showed homogeneity in primary cultures, and their morphology, karyotype and stemness property remained unaltered in long-term cultures [88]. Moreover, these cells, compared to BM-MSCs, have a higher proliferation index that remains constant in long-term culture by the regular activation of the telomerase enzyme [55]. Many studies reported that hGMSCs, thanks to their differentiative and regenerative potential, also play a pivotal role in bone regeneration, tendon repair, periodontal tissue restoration, and wound healing [89,90]. However, their osteogenic differentiation potential is reduced if compared to BM-MSC [91]. As reported earlier, hGMSCs also show important immunomodulatory capabilities, which make these cells valid alternatives for the treatment of inflammatory and autoimmune diseases [92]. Furthermore, hGMSCs are not tumorigenic. Unlike BMSCs, which can lead to the growth of tumors after their in vivo application, the therapeutic use of hGMSCs does not determine genotoxicity and oncogenesis. The use of hGMSCs can be considered potentially safer than BM-MSCs [93]. Despite their potential and countless benefits, hGMSCs have some limitations. One of the limits of hGMSCs is their reduced potential compared to the pluripotency of stem cells with embryonic origins, but on the other hand, their use is not linked to ethical obstacles. Instead, the disadvantages related to the use of these cells are due to the variability of their properties in relation to donors. Factors such as a donor’s health, age, and lifestyle can alter the properties of these cells, reducing their regenerative potential [94].

## 8. Conclusions

To date, hGMSCs are widely used as a model for in vivo and in vitro studies. In recent years, the regenerative capacity of hGMSCs and their innumerable properties, such as self-renewal, anti-inflammatory, and immunomodulatory capacities, have led many scientists to focus on their great potential in the regenerative medicine field. In addition, EVs derived from hGMSCs and their secretome could represent a promising multifactorial therapeutic approach.

## Figures and Tables

**Table 1:** hGMSCs multipotency.

Differentiation	Differentiation Markers	Differentiation Medium	Differentiation Time	Ref.
Adipogenic	PPARγ2, FABP4	DMEM + 10% FBS, 10 µmol/L dexamethasone, 10 nmol/L 3-isobutyl-1-methylxanthine, 5 µg/mL insulin and 60 µmol/L indomethacin	28 days	[13,14]
Osteogenic	RUNX2, OCN, OPN	DMEM + 15% FBS, 10 nM dexamethasone, 10 mM glycerophosphate and 0.05 mM ascorbic acid	21 days	[15]
Endothelial	CD31	Endothelial growth medium + 2%FBS and 50 ng/mL of VEGF	7 days	[11]
Neurogenic	GFAP, MAP2, S100, nestin, β-tubulin III	Medium 1: DMEM + 5 ng/mL FGF-2, 5 ng/ ml−1 nerve growth factor, 2 ng/mL epidermal growth factor, 10 μM hydrocortisone and 0.1 mM 3-isobutl-1-methylxanthine Medium 2: DMEM + 0.5 μM retinoic acid in 3% FBS	4 days Medium 1 3 days Medium 2	[16]
Chondrogenic	COL2A1, ACAN	Chondrogenic Induction medium supplemented with α-MEM +1% FCS, 50 nM ascorbate-2-phosphate, 10 ng/mL TGF-b1, 6.25 mg/mL insulin and 1% antibiotic/antimycotic.	35–42 days	[17]
Odontogenic	ALP, OPN, BSP, DMP-1	Odontogenic differentiation medium + 100 nmol/L dexamethasone, 50 mg/mL ascorbic acid, and 10 mmol/L β-glycerophosphate	14 days	[18,19]
Myogenic	MF20, Myf5, MyoD	α- MEM + 15% FBS, 2 mM L-gluta, 100 nM Dex, 100 µM ascorbic acid, 2 mM sodium pyruvate, 100 U/mL penicillin, 100 µg/mL streptomycin and a cocktail of 10 µM forskolin (FSK), MeBIO, and 10 ng/mL recombinant h bFGF	28 days	[20]

**Table 2:** Oral mesenchymal stem cells.

Name	Tissue Origin	Lineage Differentiation	Cell Surface Markers	Ref.
hPDLSCs	Periodontal ligament	Osteoblasts, adipocytes, chondrocytes, and cementoblasts	Positive: STRO-1, STRO-3, CD13,CD29, CD44,CD90, CD146, CD105, CD106, and CD166 Negative: CD14, CD34, CD45, CD38, CD54, and HLA-DR	[32]
hDPSCs	Dental Pulp	Osteoblasts, chondrocytes, myocytes, neuronal cells, adipocytes, and cardiomyocytes	Positive: CD29, CD44, CD73, CD105. Negative: CD14, CD34, and CD45	[33]
hSHEDs	Exfoliated deciduos teeth	Odontoblasts, endothelial cells, neural cells, osteoblasts, chondrocytes, adipocytes, and myocytes	Positive: Oct4, CD13, CD29, CD44, CD73, CD90, CD105, CD146, and CD166. Negative: CD14, CD34, or CD45	[34]
hGMSCs	Gingiva	Adipocytes, chondrocytes, osteocytes, endothelial cells, neurons, odontoblasts, and myocytes	Positive: CD44, CD73, CD90, CD105, SSEA-4, STRO-1, CD146, CD166, and CD271. Negative: CD14, CD19, CD34, and CD45.	[20,35,36]
hSCAPs	Apical papilla	Osteoblasts, odontoblasts, neural cells, adipocytes, chondrocytes, and hepatocytes	Positive: STRO-1 and CD146, CD13, CD24, CD29, CD44, CD49, CD51, CD56, CD61, CD73, CD90, CD105, CD106, CD166, NOTCH3, and vimentin. Negative:CD14, CD18, CD34, CD45, CD117, and CD150.	[37]
hDFSCs	Dental Follicle	Osteoblasts, cementoblasts, adipocytes, and neuron-like cells	Positive STRO-1, CD44, CD29, CD 90, CD73 and CD105, and negative CD34, CD45 and CD117.	[38]

**Table 3:** Immunomodulatory properties of hGMSCs.

Target	Mechanism of Action	Effects	Ref.
Monocytes/ macrophages	Promotion of macrophage polarization toward the M2 type.	Increment of anti-inflammatory cytokines IL-6, IL-10 and arginase-1. Reduction in pro-inflammatory cytokines TNF-α, IL-12, and IL-1β.	[45]
Dendritic cells (DCs)	Inhibition of DCs differentiation, maturation and functionality. Reduction in DCs’ ability to activate T cells.	Increment of PD-L1 and pro-inflammatory cytokines as IL-12. Reduction in DCs markers CD11c and CD80.	[46]
T cells	Inhibition of T cell proliferation by apoptosis and cell-cycle arrest.	Increment of PGE2 and IL-10. Reduction in proinflammatory cytokines IFN-γ, IL-17.	[47,48]
B cells	Arrest of B cells in the G_0_/G_1_ phase of the cell cycle by inhibiting cyclin D2 and upregulating p27. (the effect of hGMSCs on B-cell viability and proliferation is related to the proliferative state of B cells)	Block gene expression involved in chemokine signaling and reduce total IgG and IgM autoantibodies.	[49]
Mast cells (MC)	Inhibition of MC degranulation and release of pro-inflammatory cytokines.	Reduction in MC markers such as CD 117 and reduction in pro-inflammatory cytokines TNF-α and IL-4.	[46]

**Table 4:** hGMSCs application in regenerative medicine.

Application	Scaffold	Aim	Model	Spp.	Exp.	Finding	Ref.
Oral craniofacial disordes	Matriderm collagen scaffold seeded with hGMSCs	Evaluate the osteoconductive activity of Matriderm collagen scaffolds with hGMSCs	Periodontal patients with jawbone resorption	Hu	in vitro	Matriderm improves hGMSCs cell growth and osteo-differentiation	[69]
Oral craniofacial disordes	3D printing biomaterials poly(lactide) (3D-PLA) seeded with hGMSCs and/or EVs	Evaluate the role of 3D printing PLA scaffold seeded with hGMSCs and/or EVs in bone regeneration	Calvaria defect	Rt	In vitro In vivo	3D printed PLA enriched with hGMSCs and EVs promote the Osteo- angiogenesis	[21]
Oral craniofacial disordes	Collagen membranes loaded with hGMSCs- CM or hPDLSC- CM	Compared the effects of hGMSCs-CM and hPDLSCs-CM on periodontal regeneration	Periodontal defect	Rt	In vitro In vivo	hGMSCs- CM and hPDLSCs- CM transplant improve periodontal regeneration	[56]
Oral craniofacial disorders	hGMSCs injection	Evaluate the role of transplanted hGMSCs in the regulation of lipid metabolism and inflammation in hyperlipidemic mice with periodontitis	Periodontal defect	Mu	in vivo	hGMSCs injections led reduce hyper- lipidemia, inflammation and promote periodontal tissue restoration in hyperlipidemic mice with periodontitis	[70]
Oral craniofacial disorders	Alginate-based adhesive hydrogel encapsulating hGMSCs	Evaluate the effect, the functionality and the ability of Alginate-based adhesive hydrogel seeded with hGMSCs, to promote bone tissue regeneration	Peri-implantitis diseases	Mu	in vivo	Alginate-based adhesive hydrogel /hGMSCs, promotes bone regeneration in craniofacial tissue	[71]

**Table 5:** hGMSCs application in regenerative medicine.

Application	Scaffold	Aim	Model	Spp.	Exp.	Finding	Ref.
Oral craniofacial disorders	hGMSCs from inflamed and healthy gingival tissue seeded into perforated collagen-coated polytetra-floro-ethylene (PTFE)	Compare the in vitro ability of hGMSCs, isolated from healthy and inflamed gingiva, to grow and migrate through microperforated membranes	Periodontal defect	Hu	In vitro	No significant differences in grow and migration ability of hGMSCs isolated from heathy and inflamed gingiva	[72]
Oral craniofacial disorders	Pre differentiated hGMSCs (dGMSCs) combinated with hydrogel scaffold PuraMatrix (PM) and/or BMP2	Evaluate the ability of dGMSCs to differentiate into osteocytes when combinated with PM and/or BMP2	Maxillary alveolar bone defect	Rt	in vivo	dGMSCs with PM and low doses of BMP2 promote bone regeneration	[73]
Skin disorders	3D-printed medical-grade poly- caprolactone (mPCL) dressing seeded with hGMSCs	Evaluate the effect of the construct mPCL/hGMSCs in the wound closure.	Splinted excisional wound	Rt	In vitro In vivo	mPCL/hGMSCs accelerates the wound closure and reduces scar formation	[74]
Skin disorders	IL-1β–primed hGMSCs engraft	Compare the role of 1L-1β-primed hGMSCs and naive MSCs (NV-MSCs) in wound healing and epidermal engraftment	Full- thickness excisional wound	Mu	In vivo In vitro	IL-1β-primed hGMSCs promotes cell migration, generates dermal- epidermal junctions and drecreases inflammation in vitro.IL-1β-primed hGMSCs promotes epidermal substitute engraftment in vivo.	[75]

**Table 6:** hGMSCs application in regenerative medicine.

Application	Scaffold	Aim	Model	Spp.	Exp.	Finding	Ref.
Bone regeneration	bovine pericardium collagen membranes (BioRipar, BioR) seeded with hGMSCs	Evaluate the role of ascorbic acid (AS) addition to BioR/hGMSCS	Bone diseases	Hu	In vitro	The addition of AS to BioR/hGMSCs improves osteogenesis	[76]
Bone regeneration	hBMSCs or hGMSCs loaded in a NanoBone scaffold	Compare the regenerative potential of hBMSCs and hGMSCs seeded in a NanoBone scaffold	Critical- sized tibial bone defects	Rb	in vivo	The application of hGMSCs and hBMSCs loaded on the NanoBone scaffold improves bone regeneration	[77]
Bone regeneration	hGMSCs/ hGMSCs-Exo injection	Compare the immunomodulatory effects of hGMSCs-Exo and hGMSCs	Collagen-induced arthritis (CIA)	Mu	In vitro In vivo	Both hGMSCs-Exo and hGMSCs reduce inflammation and bone erosion	[64]
Nerve regeneration	Gelfom embedded with hGMSCs-EVs	Evaluate the effects of hGMSCs-EVs on peripheral nerve regeneration	Crush- injured sciatic nerves	Mu	in vivo	hGMSCs- EVs support peripheral nerve regeneration through the activation of c-JUN- governed repair phenotype of Schwann cells	[78]
Nerve regeneration	hGMSCs–Moringin (MOR) treatment	Evaluate the role of hGMSCs, pretreated with nanostructured liposomes enriched with MOR, in regeneration, inflammation and apoptosis processes	Spinal cord injury (SCI)	Mu	In vitro	MOR-treated hGMSCs reduce the expression of pro- inflammatory cytokines and restore the morphology of spinal cord.	[79]

**Table 7:** hGMSCs application in regenerative medicine.

Application	Scaffold	Aim	Model	Spp.	Exp.	Finding	Ref.
Nerve regeneration	hGMSC-EVs combined with biodegradable chitin conduits	Evaluate the effects of Chitin conduits/hGMSC-EVs on peripheral nerve regeneration	Sciatic nerve defect	Rt	In vitro In vivo	Chitin conduits/ hGMSC-EVs enhance the amount and the size of nerve fibers and improves myelin formation	[80]
Nerve regeneration	hGMSC seeded into chitosan/βglycero- phosphate hydrogel in addition with growth factor and metformin	Evaluate the metformin ability to promote hGMSCs differentiation towards the neuronal lineage, in a growth environment of chitosan hydrogel	Neurological diseases	Hu	In vitro	Metformin has no effect on multi- directional differentiation potential of hGMSCs	[81]
Inflammatory diseases	hGMSC injection	Evaluate the anti- inflammatory effetcs of hGMSCs and their therapeutic effect on inflammatory diseases	Inflam. bowel disease (IBD)	Mu	in vivo	hGMSC treatment increases the numbers of anti- inflammatory cytokines and reduces the production of pro- inflammatory cytokines	[82]
Inflammatory diseases	hGMSC injection	Evaluate the role of CD39 in the attenuation of hGMSCs- mediated acute graft-versus-host disease	Acute graft- versus-host disease (GVHD)	Mu	in vivo	hGMSCs ability to attenuate GVHD is related to the upregulation and differentiaiton of Tregs mediated by CD39 pathway	[83]

## Data Availability

Not applicable.
